# Evidence that blood-CSF barrier transport, but not inflammatory biomarkers, change in migraine, while CSF sVCAM1 associates with migraine frequency and CSF fibrinogen

**DOI:** 10.1111/head.14088

**Published:** 2021-03-16

**Authors:** Robert P. Cowan, Noah B. Gross, Melanie D. Sweeney, Abhay P. Sagare, Axel Montagne, Xianghong Arakaki, Alfred N. Fonteh, Berislav V. Zlokovic, Janice M. Pogoda, Michael G. Harrington

**Affiliations:** 1 Department of Neurology, Stanford University, Palo Alto, CA, USA; 2 Neurosciences, Huntington Medical Research Institutes, Pasadena, CA, USA; 3 Department of Physiology and Neuroscience, Zilkha Neurogenetic Institute, Keck School of Medicine, University of Southern California, Los Angeles, CA, USA; 4 Cipher Biostatistics & Reporting, Reno, NV, USA

**Keywords:** albumin quotient, chronic migraine, cytokines, headache frequency, ictal migraine, soluble vascular cell adhesion molecule-1

## Abstract

**Objective:**

Our objective is to explore whether blood–cerebrospinal fluid (CSF) barrier biomarkers differ in episodic migraine (EM) or chronic migraine (CM) from controls.

**Background:**

Reports of blood–brain barrier and blood–cerebrospinal fluid barrier (BCSFB) disruption in migraine vary. Our hypothesis is that investigation of biomarkers associated with blood, CSF, brain, cell adhesion, and inflammation will help elucidate migraine pathophysiology.

**Methods:**

We recruited 14 control volunteers without headache disorders and 42 individuals with EM or CM as classified using the International Classification of Headache Disorders, 3rd edition, criteria in a cross-sectional study located at our Pasadena and Stanford headache research centers in California. Blood and lumbar CSF samples were collected once from those diagnosed with CM or those with EM during two states: during a typical migraine, before rescue therapy, with at least 6/10 level of pain (ictal); and when migraine free for at least 48 h (interictal). The average number of headaches per month over the previous year was estimated by those with EM; this enabled comparison of biomarker changes between controls and three headache frequency groups: <2 per month, 2–14 per month, and CM. Blood and CSF biomarkers were determined using antibody-based methods.

**Results:**

Antimigraine medication was only taken by the EM and CM groups. Compared to controls, the migraine group had significantly higher mean CSF-blood quotients of albumin (Q_alb_: mean ± standard deviation (SD): 5.6 ± 2.3 vs. 4.1 ± 1.9) and fibrinogen (Q_fib_ mean ± SD: 1615 ± 99.0 vs. 86.1 ± 55.0). Mean CSF but not plasma soluble vascular cell adhesion molecule-1 (sVCAM-1) levels were significantly higher in those with more frequent migraine: (4.5 ng/mL ± 1.1 in those with <2 headache days a month; 5.5 ± 1.9 with 2–14 days a month; and 7.1 ± 2.9 in CM), while the Q_fib_ ratio was inversely related to headache frequency. We did not find any difference in individuals with EM or CM from controls for CSF cell count, total protein, matrix metalloproteinase-9, soluble platelet-derived growth factor receptor β, tumor necrosis factor-alpha, interferon-gamma, interleukin (IL)-6, IL-8, IL-10, or C-reactive protein.

**Conclusions:**

The higher Q_alb_ and Q_fib_ ratios may indicate that the transport of these blood-derived proteins is disturbed at the BCSFB in persons with migraine. These changes most likely occur at the choroid plexus epithelium, as there are no signs of typical endothelial barrier disruption. The most striking finding in this hypothesis-generating study of migraine pathophysiology is that sVCAM-1 levels in CSF may be a biomarker of higher frequency of migraine and CM. An effect from migraine medications cannot be excluded, but there is no known mechanism to suggest they have a role in altering the CSF biomarkers.

## INTRODUCTION

Leading theories propose that cortical spreading depression or meningeal irritation activate the trigeminovascular system and give rise to the major symptoms of migraine.^1–4^ Although it has been proposed that events may start in the hypothalamus and/or the brainstem,^2,5–7^ it remains a “near complete mystery of how a migraine starts”.^8^ This gap in knowledge limits the understanding of how internal and external events trigger migraine in susceptible individuals and leaves uncertain the locations at which successful therapy acts.

Interoceptive signaling from blood to the brain is exquisitely regulated in the neurovascular unit at the blood-brain barrier (BBB)^9^ and in the choroid plexus epithelium at the blood-cerebrospinal fluid barrier (BCSFB).^10^ Exceptions to these barriers include the vascular organ of the lamina terminalis, subfornical organ, pineal body, area postrema, neurohypophysis, median eminence (excluding the subcommissural organ),^11^ and trigeminal ganglion.^12^ Severe barrier disruption is easily recognized in conditions such as meningitis or hemorrhage,^13^ but studies of signaling across barriers between blood, cerebrospinal fluid (CSF), and brain in migraine provide inconsistent findings.

For example, PET imaging has shown that the migraine medication dihydoergotamine does not enter the brain,^14^ and no gadolinium crosses into the CSF in dynamic contrast-enhanced MRI in migraine;^15^ conversely, BBB breakdown was demonstrated in hemiplegic migraine.^16,17^ Recently, inflammation in the occipital cortex in migraine with visual aura was reported.^18^ A leak has been inferred from nonspecific blood surrogates of the BBB^19^ and in animal model studies.^20,21^ These inconsistent findings leave some fundamental questions unresolved, including if, how, and where endogenous migraine triggers or therapeutic medications access the brain.

We hypothesized that BCSFB biomarkers would diverge across different migraine states and therefore might help to identify and localize any disturbance. To explore this strategy, we recruited volunteers with different susceptibility to migraine. Most individuals live without migraine, yet one in six Americans range from infrequent episodic to chronic migraine (CM).^22^ Individuals with chronic headache are less resilient to triggers, with fluctuations of sensory allodynia, cognitive decline, reduced response to rescue treatment, and severe decrease in life quality.^23^ To survey this migraine spectrum, we compared blood and CSF biomarkers from study participants with different migraine frequencies and from controls without any migraine history.

## METHODS

### Study design

Our cross-sectional design at two collaborating headache research clinics recruited migraine and healthy control study participants using the same protocol. Our requirement for a CSF and blood collection only for research, without any clinical indication, caused the duration of recruitment to range from 2004 to 2016. In this report, we present the primary analysis of these data undertaken in 2018–2019.

### Study participants

Study participants were recruited from either of the headache research clinics. Inclusion criteria were age 18–80 years with episodic migraine (EM) or CM based on the International Classification of Headache Disorders, 3rd edition (ICHD-3) criteria.^24^ Each study participant estimated their average monthly headache frequency over the previous year. At the time of CSF and blood collection from EM participants, the interictal state was defined as no headache (0 of 0–10 scale) for >48 h at the time of CSF collection; the ictal state was defined as severe and typical migraine between 2 and 8 h of onset, with the pain scored at 6/10 or higher, before any use of rescue medication and without any change of daily medication over the preceding month. Control participants, selected with similar age, sex, and socioeconomic background as those with migraine, were recruited from both headache clinics over the same time period. These volunteers were either family members of clinic participants or had gained interest in the study from the local community. Inclusion for controls was then based on our finding no primary headache disorder after administering the same medical assessment as that given to those with migraine. Blood and lumbar CSF were collected between the hours of 8 a.m. and 3 p.m. at both locations.

### Exclusion criteria

Participants with other chronic headache conditions, other active or untreated medical conditions, or contraindications for lumbar puncture were excluded.

### Procedures

We used the same protocols for blood and CSF at both recruitment sites. Venous blood from the antecubital fossa was collected in potassium ethylenediaminetetraacetic acid tubes, centrifuged at 3000 g for 3 min, and plasma was aliquoted and stored at −80°C until thawed for assay. CSF was collected using standard lumbar puncture (~20 mL), 500 μL of the fluid was used for cell counts and protein assay, and the bulk of fluid was centrifuged at 800 g for 3 min, the supernatant aliquoted, and stored at −80 °C until thawed for assay.

### CSF and plasma analyses

All assays were undertaken between 2018-2019 from samples accumulated and stored over the long duration necessary for their acquisition, all assays were undertaken at the same time, between 2018 and 2019. Each assay run included samples from all clinical groups, and the technician performed the analysis without knowledge of any clinical classification. We have validated the following in-house measures and commercial kits used for all analytes in our prior publications.

### Cell counts and protein assay

Cells were counted in a hemocytometer with trypan blue. Total protein concentrations were determined using the fluorescent Quant-iT™ protein assay kit (Invitrogen/Molecular Probes, Eugene, OR) with bovine serum albumin (0–500 ng/mL) as a standard for quantification. Fluorescence (excitation at 470 nm and emission at 570 nm) was measured using a Gemini XPS Dual-Scanning Microplate Spectrofluorometer and data were analyzed using SoftMax® Pro software (Molecular Devices, Sunnyvale, CA).

### Albumin and fibrinogen quotients

An enzyme-linked immunosorbent assay (ELISA) was used to determine CSF and plasma albumin (catalog # E-80AL, Immunology Consultant Laboratories, Portland, OR). CSF fibrinogen levels were quantified using fibrinogen ELISA (catalog # E-80FIB, Immunology Consultant Laboratories, Portland, OR). The CSF/plasma albumin or fibrinogen quotients were calculated as Q_alb_ = CSF albumin (mg/L)/plasma albumin (g/L) and Q_fib_ = CSF fibrinogen (mg/L)/plasma fibrinogen (g/L).

### Quantitative western blotting of soluble platelet-derived growth factor receptor-β (sPDGFRβ) in human CSF, a pericyte injury marker

The quantitative western blot analysis was used to detect sPDGFRβ in human CSF (ng/mL), as we previously reported.^26,27^ Briefly, CSF samples and recombinant human PDGFRβ carrier-free protein (Cat. No. 385-PR-100/CF, R&D Systems) were subjected to 4–12% Bis-Tris SDS/PAGE gel electrophoresis (Thermo Scientific) for 2 h at 150 V and subsequently transferred to a nitrocellulose membrane. The membrane was blocked for 1 h with a superblock blocking buffer (Cat. No. 37537, Thermo Scientific). The membrane was incubated with a primary antibody for PDGFRβ (Cat. No. AF1042, R&D Systems) overnight, then incubated with donkey anti-goat IgG secondary antibody (Cat. No. A15999, 1:5000 dilution, Thermo Scientific) for 1 h at room temperature, treated with Western ECL detection solution (Cat. No. 34075, Thermo Scientific), exposed to CL-Xposure film (Cat. No. 34091, Thermo Scientific), and developed in an X-Omat 3000 RA film processor (Kodak). Images were acquired, and densitometry analysis was performed using NIH Image J software.

### Plasminogen

ELISA was used to determine CSF plasminogen levels (catalog # E-80PMG, Immunology Consultant Laboratories, Portland, OR).

### Endothelial markers

The Meso Scale Discovery (MSD) assay was used to determine soluble intracellular adhesion molecule-1 (sICAM1) and soluble vascular cell adhesion molecule-1 (sVCAM1) levels (catalog # K15198D, MSD, Rockville, MD).

### Inflammatory markers

The MSD assay (catalog # K15049G, MSD, Rockville, MD) was used to determine interleukin (IL)-2, 6, 8, and 10, tumor necrosis factor-alpha (TNFα) and interferon-gamma (IFNγ) levels. The MSD assay (catalog # K15198D, MSD, Rockville, MD) was used to determine C-reactive protein (CRP) levels in CSF and plasma.

### Matrix metalloproteinase-9 (MMP-9)

The active form of MMP-9 was determined using ELISA (catalog # 72017, AnaSpec, Fremont, CA).

### Statistical analysis

There was no formal sample size calculation as there were no a priori effect sizes on which to power the planned analyses. Thus, the sample size was based on CSF-blood availability.

Because samples from the migraine population were taken during both ictal and interictal states, analyses were done using mixed model repeated measures (MMRM) with compound symmetry covariance matrix. Biomarkers were the dependent variables and the migraine group was the fixed independent variable. Age and sex were included as covariates. Data transformations were used to satisfy model assumptions of normality and variance heteroscedasticity of residuals, and extreme outlying values were investigated to determine whether they should be excluded from analyses (footnoted in each table). For inflammatory biomarkers, observations < lower limit of detection (LLD) were imputed with the value LLD/2 (20/67 observations). Tests for trend over groups defined by migraine frequency for permeability, BBB, and cell adhesion biomarkers were done by modeling the frequency group as a continuous variable.

All analyses were preplanned. Statistical testing was done using two-sided 0.05 significance levels without adjustment for multiplicity. SAS version 9.4 (SAS, Inc., Cary, NC) was used for most statistical analyses. Prism v8 was used for the summary of demographic and baseline characteristics (Table 1) and for Spearman correlations and confidence intervals shown in the Figure S1. Numeric variables were summarized as means and SDs along with marginal means and standard errors (SEs) to account for covariate adjustment and multiple observations per subject. Categorical variables were summarized as frequencies and percentages.

## RESULTS

### Study population

In total, 76 individuals were screened for the study and were self-selected, as none declined to have the lumbar puncture. A total of 56 (74%) screened individuals participated in the study: 14 controls and 42 met criteria for migraine, including 20 without aura, 6 with aura, and 16 with CM. Eleven of those with EM participated in both ictal and subsequent interictal states. None of the interictal EM or CM participants had migraine or post-LP headache in the 48-h subsequent to fluid collection. Group demographics and baseline characteristics are shown in Table 1. CSF cell counts were normal (no evidence of traumatic tap), no abnormal cells were seen, and total CSF protein was in the normal range in all groups.

### Fluctuations in BCSFB biomarkers distinguish migraine from controls

We measured common BCSFB biomarkers^28^ for evidence of leakage from the blood (Table 2). The albumin quotient (Q_alb_), fibrinogen quotient (Q_fib_) and CSF plasminogen levels were significantly higher in persons with migraine than in controls, consistent with increased transport from blood to CSF. We did not find significant differences between migraine and control participants in blood fibrinogen levels (1.41 mg/mL ± 0.54 migraine group vs. 1.64 mg/mL ± 0.54 controls). MMP-9 and sPDGFRβ levels were similar between the migraine and control groups. sVCAM1 levels were descriptively higher in CSF from the migraine group but did not reach significance for this sample size. The levels of inflammatory markers in both CSF and plasma (Table 3) did not reflect substantial inflammation. The IL-10 levels were significantly lower in migraine.

### Response to ictal, interictal, or more frequent migraine

In exploring the dynamic effects that different migraine states might have on the BCSFB, we observed no significant differences between ictal and interictal migraine (Table 4). The Q_fib_ ratio was negatively related and the sVCAM1 levels were positively related to average monthly headache frequency. The sVCAM1 relationship was significant both with and without controls included in the analysis (Table 5 and Figure 1). Differences observed for other CSF biomarkers were not statistically significant.

## DISCUSSION

We investigated migraine pathophysiology through biomarkers of BCSFB, cell adhesion, and inflammation, and found evidence of distinctly altered transport of albumin and fibrinogen in migraine based on the Q_fib_ and, to a lesser extent, the Q_alb_ quotient. The Q_fib_, adjusted for age and sex, did not change significantly with the average monthly headaches, suggesting that this BCSFB change is a measure of overall migraine predisposition and not a response to the frequency of migraine. Interestingly, observed CSF sVCAM1 levels in EM were descriptively higher in those with more headaches per month, and the trend was statistically significant across all frequency groups, both with and without controls, which implies a migraine-responsive role for sVCAM1.

### Does the BCSFB differ in migraine and does it matter?

The measure of Q_alb_ is established as a sensitive indicator of blood–CSF disruption.^29^ Neither albumin nor fibrinogen is expressed in the brain (http://www.proteinatlas.org/ENSG00000171560-FGA/tissue). Our data suggest that transport of albumin, fibrinogen, and plasminogen is altered at the BCSFB in migraine, though we do not yet know the mechanism. This, and the lack of altered cells or total protein in CSF, suggest a specific and selective process is altered in migraine, rather than a molecular weight-based filtrate; there is no uncontrolled barrier disruption that one associates with a “leak.”

In considering the consequences of altered blood-CSF permeability in migraine, albumin is a major transporter of calcium, hormones, and lipids, with known roles in migraine.^1,30^ Increased albumin transport to CSF of any of these molecules in migraine would have significant consequences. Though fibrinogen has not been found as a genetic risk factor for migraine compared to controls,^31,32^ higher fibrinogen levels in CSF may reflect a susceptibility to the triggering of migraine. Most relevant to the fluctuating brain state of migraine is a report of higher CSF fibrinogen levels in major depressive disorder,^33^ a condition comorbid with migraine.

### Where is the blood-CSF transport altered?

Biomarkers cannot distinguish barrier locations unequivocally; however, the altered permeability manifest in our data more likely occurs at the epithelial cells of the choroid plexus than at the endothelial cells of the brain’s neurovascular unit (NVU). Our data support that there is no NVU breakdown, consistent with a Danish group that reported no NVU leak in migraine with dynamic contrast-enhanced MRI experiments.^15^ It remains unresolved how systemic signals modulate migraine, including how and where endogenous migraine triggers or therapeutic medications access the brain. Our study supports an altered activity in the choroid plexus epithelium and circulating CSF in migraine. Since blood-borne migraine therapeutics can access the basal side of the choroid plexus epithelium, we propose this is a vital locus to investigate their site of action.

### Why do sVCAM1 levels differ with headache frequency?

CSF sVCAM1 has not been studied before in migraine, to our knowledge. Plasma sVCAM1 levels are elevated in aging along with evidence that brain endothelial cell VCAM1 may be a treatment target for age-related neurodegeneration.^34^ However, plasma sVCAM1 levels did not differ in our migraine participants from controls. Our finding that both CSF fibrinogen and sVCAM1 change in migraine may arise from altered cell adhesion, since fibrinogen and plasminogen both participate in cell adhesion (https://www.uniprot.org/uniprot/P02680) and fibrinogen blocks the integrin-binding site of VCAM1 at the RGD sequence.^35^ This result raises the possibility that sVCAM1 levels increases in migraine to protect from the higher CSF fibrinogen levels we report herein. Higher sodium levels were demonstrated to stimulate VCAM1 activation,^36^ which is of particular relevance to migraine given our extensive evidence for higher CSF [Na^+^] in both preclinical and clinical migraine pathophysiology.^25,37,38^

### Is inflammation widespread in migraine?

The recent imaging evidence of inflammation at the occipital cortex in migraine with visual aura^18^ is compelling because localized changes match specific symptoms, and our study would not exclude such a localized NVU leakage. That we find no change in CSF for a wide variety of inflammatory biomarkers argues against a primary role for traditional inflammation in migraine. CSF sVCAM1 increases in inflammatory models,^39–42^ which may link the higher sVCAM1 levels we find in migraine with the frequent rescue benefit from anti-inflammatory medications such as non-steroidal anti-inflammatory agents. We found no evidence of inflammation as evaluated by normal CSF cell count, total protein, and inflammatory cytokines, and low levels of correlation between any of the cytokines with Q_alb_, Q_fib_, or CSF sVCAM1 levels (Figure S1). Overall, our varied biomarker data provide multiple, independent lines of evidence against significant inflammatory process at the BCSFB in migraine. However, our study does not evaluate transient, small, or localized brain tissue inflammation.

Limitations of this study arise from the use of recall to quantify headache frequency; the reliance on volunteers, particularly because of the invasive procedure involved; the inability to control for potential confounders; and from the exploratory nature of the study. Retrospective estimations of average monthly headache frequency are not ideal but were ascertained prior to the analysis of the relationship between headache frequency and biomarkers and differential misclassification seems unlikely. Obtaining CSF with migraine limits the number of volunteers and presents the most significant restriction for a larger cohort in this study and perhaps the most important source of bias; for example, controls may have different motivations for participating in such a study compared to individuals with migraine, and these differences may correlate with differences in biomarkers compared to the general population. Migraine medications may also have affected comparisons between migraine and control participants and between the migraine groups; however, it is difficult to assess the potential impact since nearly all individuals with migraine use these medications and controls do not. There may be other confounders affecting our findings that we either did not measure or are not measurable; the fact that the marginal means were, in some instances, quite different from the crude means ex-emplifies the effect that confounders—in this case, age and sex— can have when comparing outcomes between migraine and control participants.

We did not have a priori hypotheses to test, because it is unknown which biomarkers and what effect sizes may be clinically relevant. Therefore, the study was not powered to detect any particular differences between the migraine and control groups. We did not control for multiplicity so as not to increase the probability of type 2 error. Because of this, we used statistical significance, and lack thereof, as more of a guide for future research than for making conclusive statements; we also used descriptive summary statistics to evaluate potentially important differences. External validity will need to be sought by replication, especially with patients from different populations, and in longitudinal study. However, because our findings are in CSF not plasma samples, CSF collections will clearly limit further study in the majority of clinical settings. Nevertheless, our data justify the potential for further CSF or molecular brain imaging research in migraine. Perhaps more tractable, animal model investigations and unbiased -omics approaches are needed to understand the mechanisms and locations of the transport changes in migraine at the tissue/cell level.

## Supplementary Material

head14088-sup-0001-supinfo (1)

## Figures and Tables

**Figure:**
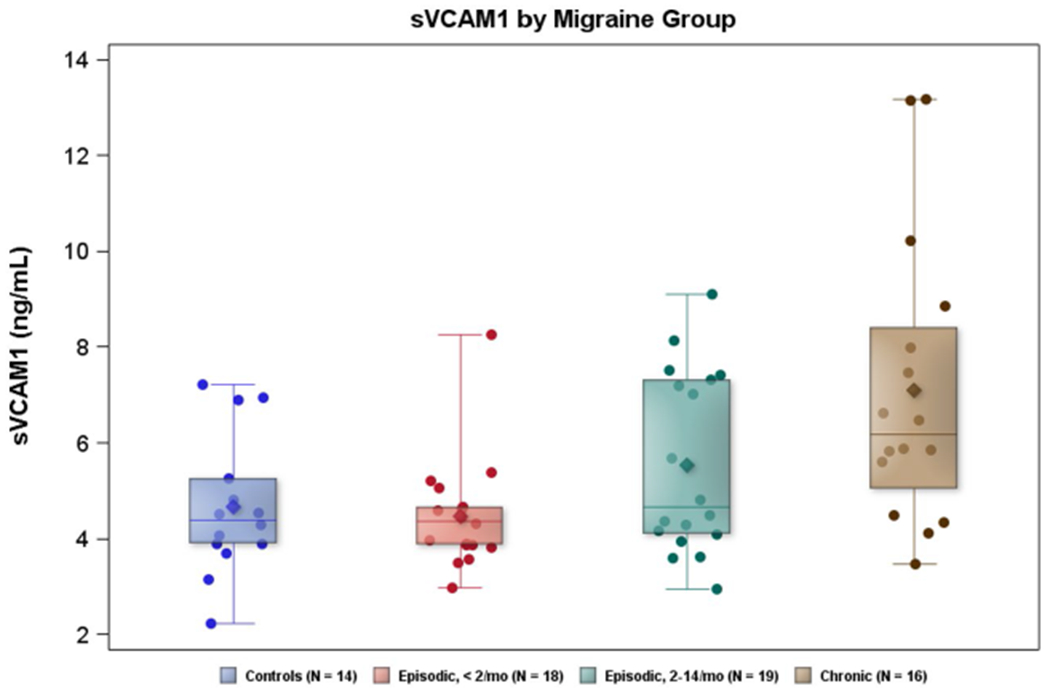
sVCAM1 levels are plotted for controls and migraine subgroups by headache frequency. Diamond in box = mean; horizontal line in box = median; box = 25th-75th percentiles; whiskers = 95% CI

**TABLE 1:** Demographics and baseline characteristics

	Controls	Interictal^a^	Ictal^a^	Chronic
Parameter	*n* = 14	*n* = 24	*n* = 13	*n* = 16
Mean (SD) age, year	40.9 (17.8)	45.2 (15.7)	45.2 (15.9)	39.6 (15.1)
Age range, year	19–75	17–77	17–77	17–65
Women, *n*(%)	10 (71.4)	19 (79.2)	10 (76.9)	15 (93.8)
Mean (SD) BMI	27.47 (6.60)	27.50 (6.24)	26.54 (5.16)	27.10 (7.74)
Current hypertension, *n*(%)	1 (7.1)	1 (4.2)	1 (7.7)	1 (6.3)
Mean (SD) CSF total protein, g/L	0.35 (0.05)	0.345 (0.07)	0.36 (0.09)	0.355 (0.07)
CSF cell count per mL	<5 (normal)	<5 (normal)	<5 (normal)	<5 (normal)

a Eleven individuals were sampled in both ictal and interictal states of episodic migraine.

**TABLE 2:** BBB, BCSFB, and cell adhesion biomarkers

Parameter	Controls (*n* = 14) Mean (SD) Marginal mean (SE)	Migraine (*n* = 42^a^) Mean (SD) Marginal mean (SE)	*p* value^b^	*p* value adj. for age and sex
Q_alb_	4.1 (1.9) 4.6 (0.6)	5.6 (2.3) 6.2 (0.4)	0.041	0.029
Q_fib_	86.1 (55.0) 111.2 (21.6)	161.5 (99.0) 187.4 (15.3)	0.007	0.001
CSF MMP-9 (pg/mL)	100.7 (27.9) 711.3 (92.8)	103.5 (29.9) 106.9 (3.8)	0.661	0.826
CSF sPDGFRβ (ng/mL)	696.4 (293.0) 711.3 (92.8)	787.4 (340.3) 803.2 (47.9)	0.422	0.494
CSF sICAM1 (ng/mL)	1.5 (0.5) 1.5 (0.2)	1.6 (0.7) 1.7 (0.1)	0.359	0.363
CSF sVCAM1 (ng/mL)	4.7 (1.5) 4.7 (0.6)	5.6 (2.3) 5.8 (0.4)	0.090	0.101
CSF plasminogen (ng/mL)	186.2 (90.4) 195.5 (29.5)	240.7 (104.7) 259.2 (20.8)	0.033	0.033
Plasma MMP-9 (ng/mL)	1.1 (0.7) 1.0 (0.2)	0.9 (0.8) 0.8 (0.2)	0.373	0.221
Plasma sICAM1 (ng/mL)	330.5 (72.1) 326.5 (28.3)	316.4 (107.9) 307.3 (18.5)	0.442	0.390
Plasma sVCAM1 (ng/mL)	470.4 (125.2) 460.3 (42.1)	487.1 (156.9) 475.6 (27.5)	0.878	0.854

a Forty-two subjects contributed to 53 observations.

b MMRM. Log-transformed data were used for CSF sICAM1, sVCAM1, and plasminogen and plasma MMP-9 and sVCAM1. Cube-root-transformed data were used for Q_alb_, Q_fib_, CSF sPDGFRβ, and plasma sICAM1. For CSF MMP-9, the analysis was done by taking the square-root transformation of the absolute value of the deviation from the median, times the sign of the deviation.

**TABLE 3:** Inflammatory biomarkers in CSF and plasma

Parameter	Controls (n = 14) Mean (SD) Marginal mean (SE)	Migraine (n = 42^a^) Mean (SD) Marginal mean (SE)	*p* value^b^	*p* value adj for age and sex
CSF IL-6 (pg/mL)	2.7 (2.3) 2.9 (0.7)	3.8 (2.6) 4.1 (0.5)	0.146	0.078
CSF IL-8 (pg/mL)	87.0 (38.6) 94.7 (14.4)	114.2 (52.1) 122.9 (10.2)	0.130	0.086
CSF IL-10 (pg/mL)	0.12 (0.1) 0.15 (0.03)	0.18 (0.1) 0.20 (0.02)	0.149	0.082
CSF TNFα (pg/mL)	0.29 (0.2) 0.33 (0.05)	0.35 (0.2) 0.38 (0.04)	0.427	0.380
CSF IFNγ (pg/mL)	0.49 (0.5) 0.50 (0.1)	0.59 (0.5) 0.58 (0.1)	0.441	0.504
CSF CRP (ng/mL)	3.3 (3.4) 2.7 (1.7)	4.2 (6.2) 3.6 (1.2)	0.470	0.665
Plasma IL-6 (pg/mL)	2.1 (1.2) 2.1 (0.5)	2.4 (1.7) 2.5 (0.4)	0.509	0.778
Plasma IL-8 (pg/mL)	12.0 (5.5) 12.6 (3.0)	16.5 (10.9) 15.8 (1.9)	0.207	0.344
Plasma IL-10 (pg/mL)	1.6 (0.9) 1.6 (0.2)	1.1 (0.4) 1.1 (0.1)	0.031	0.014
Plasma TNFα (pg/mL)	6.5 (1.6) 6.6 (0.5)	6.9 (1.7) 6.8 (0.3)	0.461	0.693
Plasma (pg/mL)	28.7 (32.4) 28.2 (6.7)	17.2 (15.6) 15.2 (4.6)	0.278	0.152
Plasma CRP (mg/L)	3.00 (3.5) 2.7 (1.0)	2.5 (3.8) 2.0 (0.7)	0.948	0.624

a Forty-two subjects contributed to 53 observations.

b MMRM. Log-transformed data were used for CSF and plasma CRP and for plasma IL-6, IL-10, and IFNγ. Cube-root-transformed data were used for CSF IL-6, IL-10, and IFNγ and for plasma IL-8. One study participant had an extreme outlying value for plasma IL-10 due to a neck injury and was therefore excluded from the analysis of plasma IL-10. Another study participant had extreme outlying values for plasma IL-6, IL-10, and TNFα and was excluded from those analyses. Another study participant had an extreme outlying value for CSF IL-8; analyses were done both with and without the outlier, resulting in the same *p* value (0.13).

**TABLE 4:** Effect of ictal versus interictal state on Q_alb_, Q_fib_, and CSF sVCAM1

	Migraine state			
Parameter	Ictal (*n* = 13) Mean (SD) Marginal mean (SE)	Interictal (*n* = 24) Mean (SD) Marginal mean (SE)	*p* value^b^	*p* value adj. for age and sex
Q_alb_	5.0 (1.8) 6.0 (0.7)	6.1 (2.3) 6.6 (0.5)	0.454	0.513
Q_fib_	167.8 (77.6) 204.1 (30.7)	190.3 (111.2) 213.2 (23.9)	0.728	0.771
CSF sVCAM1 (ng/mL)	5.1 (1.8) 5.2 (0.5)	4.9 (1.6) 4.9 (0.4)	0.105	0.326

a Eleven individuals were sampled in both ictal and interictal states of episodic migraine.

b MMRM. Cube-root transformed data were used for Q_alb_ and Q_fib_, while log-transformed data were used for sVCAM1.

**TABLE 5:** Effect of average monthly headache (HA) days on BBB, BCSFB, and cell adhesion biomarkers

		Migraine					Migraine only	
		Episodic migraine	Episodic migraine	Chronic migraine				
Parameter	Controls (*n* = 14) Mean (SD) Marginal mean (SE)	<2 per month (*n* = 18) Mean (SD) Marginal mean (SE)	2-14 per month (*n* = 19) Mean (SD) Marginal mean (SE)	(*n* = 16) Mean (SD) Marginal mean (SE)	*p* value^ab^	Trend *p* value^a,c^	*p* value^a,b^	Trend *p* value^a,c^
Q_alb_	4.1 (1.9) 4.6 (0.6)	6.0 (1.8) 6.4 (0.6)	5.5 (2.5) 6.1 (0.7)	5.2 (2.6) 6.1 (0.6)	0.130	0.399	0.477	0.222
Adjusted					0.158	0.192	0.852	0.621

Q_fib_	86.1 (55.0) 109.0 (21.5)	198.6 (108.9) 213.2 (23.2)	167.0 (92.3) 182.5 (23.5)	121.6 (85.5) 164.7 (22.9)	0.004	0.478	0.058	0.018
Adjusted					0.0045	0.125	0.304	0.120

CSF plasminogen (ng/mL)	186.2 (90.4) 198.0 (29.6)	236.1 (68.9) 250.9 (31.4)	220.9 (95.0) 238.6 (31.0)	268.3 (142.8) 290.5 (31.5)	0.147	0.074	0.639	0.844
Adjusted					0.125	0.052	0.531	0.594

CSF sPDGFRβ (ng/mL)	696.4 (293.0) 2355.9 (3455.2)	817.5 (320.4) 422.5 (3717.9)	702.8 (381.9) −527.6 (3662.9)	848.8 (313.8) 3146.3 (3694.8)	0.348	0.368	0.280	0.670
Adjusted					0.337	0.412	0.325	0.611

CSF sVCAM1 (ng/mL)	4.7 (1.5) 4.8 (0.6)	4.5 (1.1) 4.6 (0.6)	5.5 (1.9) 5.6 (0.6)	7.1 (2.9) 7.4 (0.6)	0.004	0.001	0.007	0.002
Adjusted					0.003	0.001	0.003	0.001

a Cube-root transformed data were used for Q_alb_, Q_fib_, plasminogen, and CSF sPDGFRβ, while log-transformed data were used for sVCAM1.

b Global test for group effect using MMRM.

c Test for trend over groups defined by the frequency of migraine (controls = 0 frequency) using MMR.

## Abbreviations:

BBB: blood-brain barrier
BCSFB: blood-cerebrospinal fluid barrier
CM: chronic migraine
CRP: C-reactive protein
CSF: cerebrospinal fluid
EM: episodic migraine
ICHD-3: International Classification of Headache Disorders, 3rd edition
IFNγ: interferon-gamma
IL: Interleukin
LLD: lower limit of detection
MMP-9: matrix metalloproteinase-9
NVU: neurovascular unit
Qalb and Qfib: CSF/plasma albumin or fibrinogen quotients, respectively
sICAM1: soluble intracellular adhesion molecule-1
sPDGFRβ,: soluble platelet-derived growth factor receptor-β
sVCAM-1: soluble vascular cell adhesion molecule-1
TNFα: tumor necrosis factor-alpha
VCAM-1: cell vascular cell adhesion molecule-1
