# Supplementary material for: Evidence that blood-CSF barrier transport, but not inflammatory biomarkers, change in migraine, while CSF sVCAM1 associates with migraine frequency and CSF fibrinogen
Source: Headache. Author manuscript; Available in PMC 2021 Apr 6. (PMC8023403; doi:10.1111/head.14088)
Supplement: head14088-sup-0001-supinfo (1) [file NIHMS1684323-supplement-head14088-sup-0001-supinfo__1_.docx]

**Supplementary Table**

**Number of participants who were taking at least one drug in each class of medications**

| **Drug class*** | **Controls (14) #** | **Interictal (24)** | **Ictal (13)** | **Chronic (16)** |
| --- | --- | --- | --- | --- |
| **AED** | **0** | **0** | **0** | **7** |
| **Antihypertensive** | **1** | **1** | **1** | **2** |
| **Antidepressant** | **1** | **2** | **2** | **7** |
| **NSAID** | **3** | **20** | **11** | **11** |
| **Triptan** | **0** | **22** | **12** | **11** |
| **Onabotulinum** | **0** | **0** | **0** | **9** |
| **Other** | **1** | **12** | **11** | **9** |

***, No change in medications within >48-h before fluid collection.**

**#, missing data from 1**
